# Hidden Viral Sequences in Public Sequencing Data and Warning for Future Emerging Diseases

**DOI:** 10.1128/mBio.01638-21

**Published:** 2021-08-17

**Authors:** Junna Kawasaki, Shohei Kojima, Keizo Tomonaga, Masayuki Horie

**Affiliations:** a Laboratory of RNA Viruses, Department of Virus Research, Institute for Frontier Life and Medical Sciences, Kyoto University, Kyoto, Japan; b Laboratory of RNA Viruses, Department of Mammalian Regulatory Network, Graduate School of Biostudies, Kyoto University, Kyoto, Japan; c Department of Molecular Virology, Graduate School of Medicine, Kyoto University, Kyoto, Japan; d Hakubi Center for Advanced Research, Kyoto University, Kyoto, Japan; e Division of Veterinary Sciences, Graduate School of Life and Environmental Sciences, Osaka Prefecture University, Osaka, Japan; Columbia University Medical College

**Keywords:** RNA virus, bioinformatics, molecular epidemiology, public health, virus diversity, zoonosis

## Abstract

RNA viruses cause numerous emerging diseases, mostly due to transmission from mammalian and avian reservoirs. Large-scale surveillance of RNA viral infections in these animals is a fundamental step for controlling viral infectious diseases. Metagenomic analysis is a powerful method for virus identification with low bias and has contributed substantially to the discovery of novel viruses. Deep-sequencing data have been collected from diverse animals and accumulated in public databases, which can be valuable resources for identifying unknown viral sequences. Here, we screened for infections of 33 RNA viral families in publicly available mammalian and avian sequencing data and found approximately 900 hidden viral infections. We also discovered six nearly complete viral genomes in livestock, wild, and experimental animals: hepatovirus in a goat, hepeviruses in blind mole-rats and a galago, astrovirus in macaque monkeys, parechovirus in a cow, and pegivirus in tree shrews. Some of these viruses were phylogenetically close to human-pathogenic viruses, suggesting the potential risk of causing disease in humans upon infection. Furthermore, infections of five novel viruses were identified in several different individuals, indicating that their infections may have already spread in the natural host population. Our findings demonstrate the reusability of public sequencing data for surveying viral infections and identifying novel viral sequences, presenting a warning about a new threat of viral infectious disease to public health.

## INTRODUCTION

RNA viruses have caused numerous emerging diseases; for example, it has been reported that 94% of zoonoses from 1990 to 2010 were caused by RNA viruses (1). Mammalian and avian species are especially high-risk transmission sources for zoonotic viruses because of their frequent contact with humans as livestock, bushmeat, companion, or laboratory animals (2). Additionally, the spread of viral infectious diseases in livestock animals impacts sustainable food security and economic growth (3). Thus, large-scale surveillance of RNA viral infections in these animals would help monitor infections of known and unknown viruses that can cause outbreaks in humans and domestic animals.

Metagenomic analysis can identify viruses with low bias and has contributed substantially to elucidating virus diversity for more than a decade (4). With the increase in research using metagenomic analysis, new virus species, genera, and families have been successively established by the International Committee on Taxonomy of Viruses (ICTV) (5). However, a previous study estimated the existence of at least 40,000 mammalian viral species (6), which far exceeds the number of viral species classified by the ICTV to date (5, 7). Therefore, further research is needed to understand viral diversity and prepare for future viral pandemics. The amount of transcriptome sequencing (RNA-seq) data in public databases is growing exponentially (8); however, only a few studies have examined publicly available sequencing data for viral infections (9–11). The public data are derived from samples with various research backgrounds and may contain a wide variety of viral sequences. Therefore, analyzing publicly available RNA-seq data can be an effective way to assess the spread of viral infections and discover novel viruses.

In this study, we analyzed more than 46,000 RNA-seq data to screen hidden RNA virus infections in mammalian and avian species and identified approximately 900 infections. We also discovered six nearly complete viral genomes in livestock, wild, and laboratory animals. Phylogenetic analyses showed that some of the novel viruses were closely related to human-pathogenic viruses, suggesting the potential risk of causing disease in humans. Furthermore, viral infections were identified in several individuals collected by independent studies, indicating that their infections may have already spread in the natural host population. Our findings demonstrate the reusability of public sequencing data for surveying viral infections that may present a threat to public health.

## RESULTS

### Detection of RNA viral infections hidden in public sequencing data.

**FIG 1:**
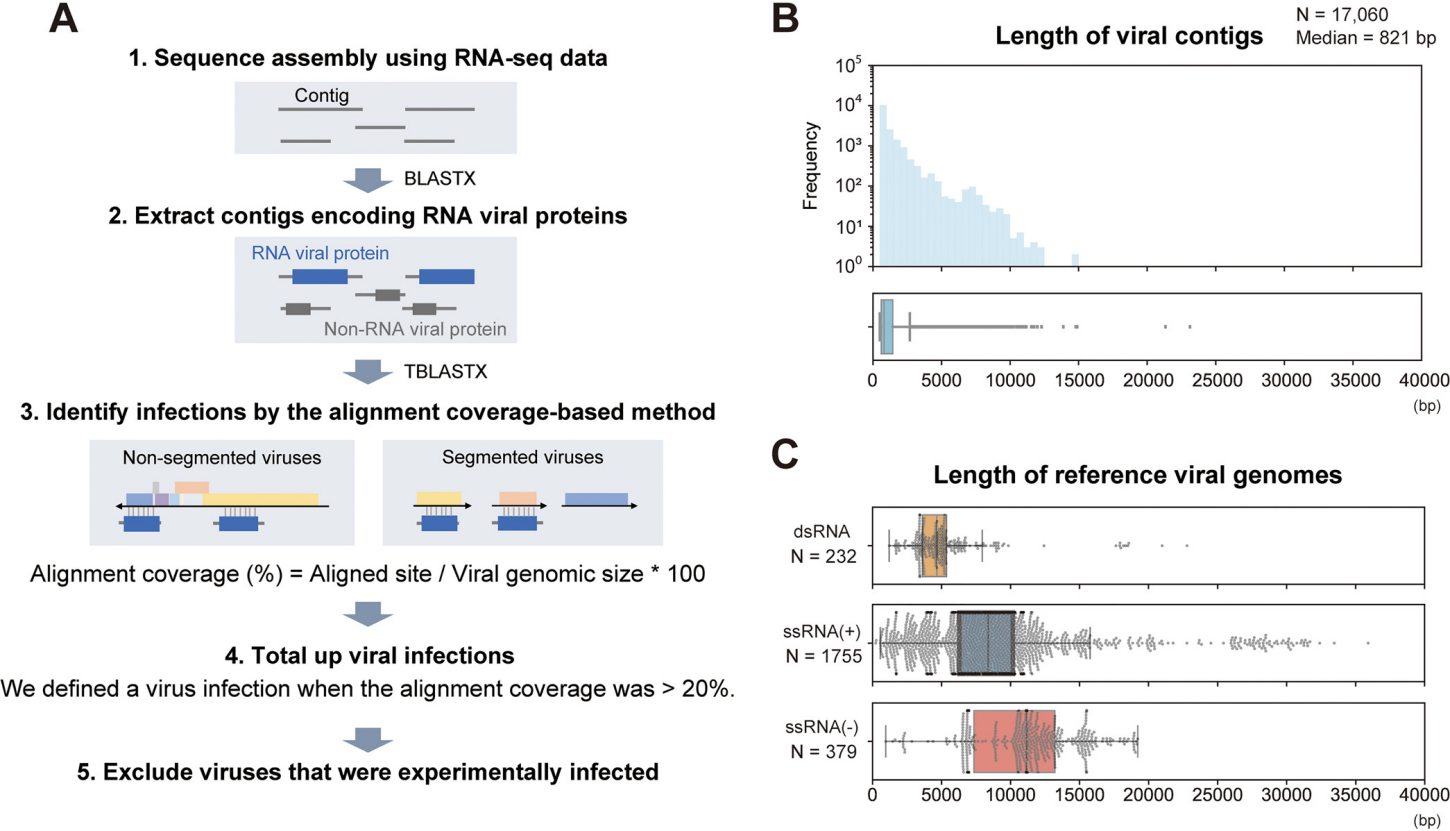
Strategy for detecting viral infections in public RNA-seq data. (A) Schematic diagram of the procedure for detecting viral infections. First, we performed *de novo* sequence assembly using publicly available mammalian and avian RNA-seq data. Second, we extracted contigs encoding RNA viral proteins by BLASTX. Third, we constructed sequence alignments by TBLASTX using the viral contigs in each RNA-seq data and reference viral genomes because most viral contigs were shorter than complete viral genomes, as shown in panels B and C. The alignment coverage is defined as the proportion of aligned sites in the entire reference viral genome. Fourth, we determined a viral infection when the alignment coverage was >20%. Finally, we totaled the infections at the virus family level after excluding experimentally infected viruses (see Materials and Methods). (B) Distributions of viral contig length: histogram (upper panel) and box plot (lower panel). The *x* axis indicates the viral contig length. Among 17,060 viral contigs, the median length was 821 bp. (C) Length of reference viral genomes. Each panel corresponds to the Baltimore classification: the upper, middle, and lower panels show double-stranded RNA (dsRNA) viruses, positive-sense single-stranded RNA [ssRNA(+)] viruses, and negative-sense single-stranded RNA [ssRNA(−)] viruses, respectively. The *x* axis indicates the viral genome size. These viral genomes were obtained from the RefSeq genomic viral database. The genomic size of segmented viruses is the sum length of all segments in a virus species.

To detect RNA viral infections in mammalian and avian RNA-seq data, we first performed *de novo* sequence assembly (Fig. 1A; see Data Set S1 in the supplemental material). We then performed BLASTX screening using contigs to extract RNA virus-derived sequences. Among 422,615,819 contigs, we identified 17,060 RNA virus-derived sequences. The median length of the viral contigs was 821 bp (Fig. 1B), which was shorter than the genomic size of RNA viruses (Fig. 1C). These results indicate that most viral contigs were detected as partial sequences of the viral genome, and several contigs may have originated from the same viral infection event. Therefore, we sought to determine the viral infections in each of the sequencing data by the alignment coverage-based method to avoid double counting (Fig. 1A; see Materials and Methods). Briefly, we constructed sequence alignments by TBLASTX using the viral contigs in each of the RNA-seq data and reference viral genomes and then calculated the alignment coverage between the viral contigs and each viral reference sequence. Here, we defined a viral infection when the alignment coverage exceeded the threshold (more than 20%). This threshold was determined using sequencing data obtained from viral infection experiments (Fig. S1; see Materials and Methods). Finally, we totaled the infections at the virus family level after excluding the viruses inoculated experimentally (Data Sets S2 and S3).

10.1128/mBio.01638-21.1 FIG S1 Validation of the alignment coverage-based method for detecting viral infections. (A) Comparison between the alignment coverage-based method and the viral read-based method using samples obtained from viral infection experiments. The *x* axis indicates alignment coverage between viral contigs in each of the RNA-seq data and the reference viral genome used for the experiments. The *y* axis indicates the total read length of the virus family used for the experiment, which was obtained from the NCBI SRA Taxonomy Analysis Tool. Light gray dots indicate samples experimentally infected with viruses, and dark gray dots indicate mock samples. *R*, Pearson’s correlation coefficient. A dotted line indicates 20% alignment coverage. (B) Changes in the true-positive and false-positive rates depending on the criteria to determine viral infections. The true-positive rate (*y* axis) indicates the number of samples experimentally infected with viruses correctly determined as the infected sample, and the false-positive rate (*x* axis) indicates the number of mock samples determined as the infected sample. Dotted lines indicate the true-positive rate (88.3%) and the false-positive rate (62.5%) when 20% alignment coverage was used as the criterion (see Materials and Methods). (C) Detection rate of viral infections depending on the viral genome size. Box plots show the distributions of alignment coverage of the viral genome with 1 to 10 kbp (green), 10 to 25 kbp (yellow), and 25 to 50 kbp (blue). Light gray dots indicate samples infected with viruses experimentally, and dark gray dots indicate mock samples. A dotted line indicates 20% alignment coverage. (D) Number of detected viral infections depending on the alignment coverage criteria. The *x* axis indicates alignment coverage used as a criterion for defining viral infections. Bar graphs show the number of detected viral infections using the criterion shown on the *x* axis. Filled colors indicate infections in samples from viral infection experiments (orange) or those in others (blue). When we used 20% alignment coverage as the criterion, a total of 1,410 viral infections were identified, including 503 experimentally infected samples. Download FIG S1, PDF file, 0.3 MB. Copyright © 2021 Kawasaki et al. 2021 Kawasaki et al. https://creativecommons.org/licenses/by/4.0/ This content is distributed under the terms of the Creative Commons Attribution 4.0 International license.

10.1128/mBio.01638-21.3 DATA SET S1 List of Sequence Read Archive run accession numbers, genome file, and sequence assembly method. Download Data Set S1, XLSX file, 1.2 MB. Copyright © 2021 Kawasaki et al. 2021 Kawasaki et al. https://creativecommons.org/licenses/by/4.0/ This content is distributed under the terms of the Creative Commons Attribution 4.0 International license.

**FIG 2:**
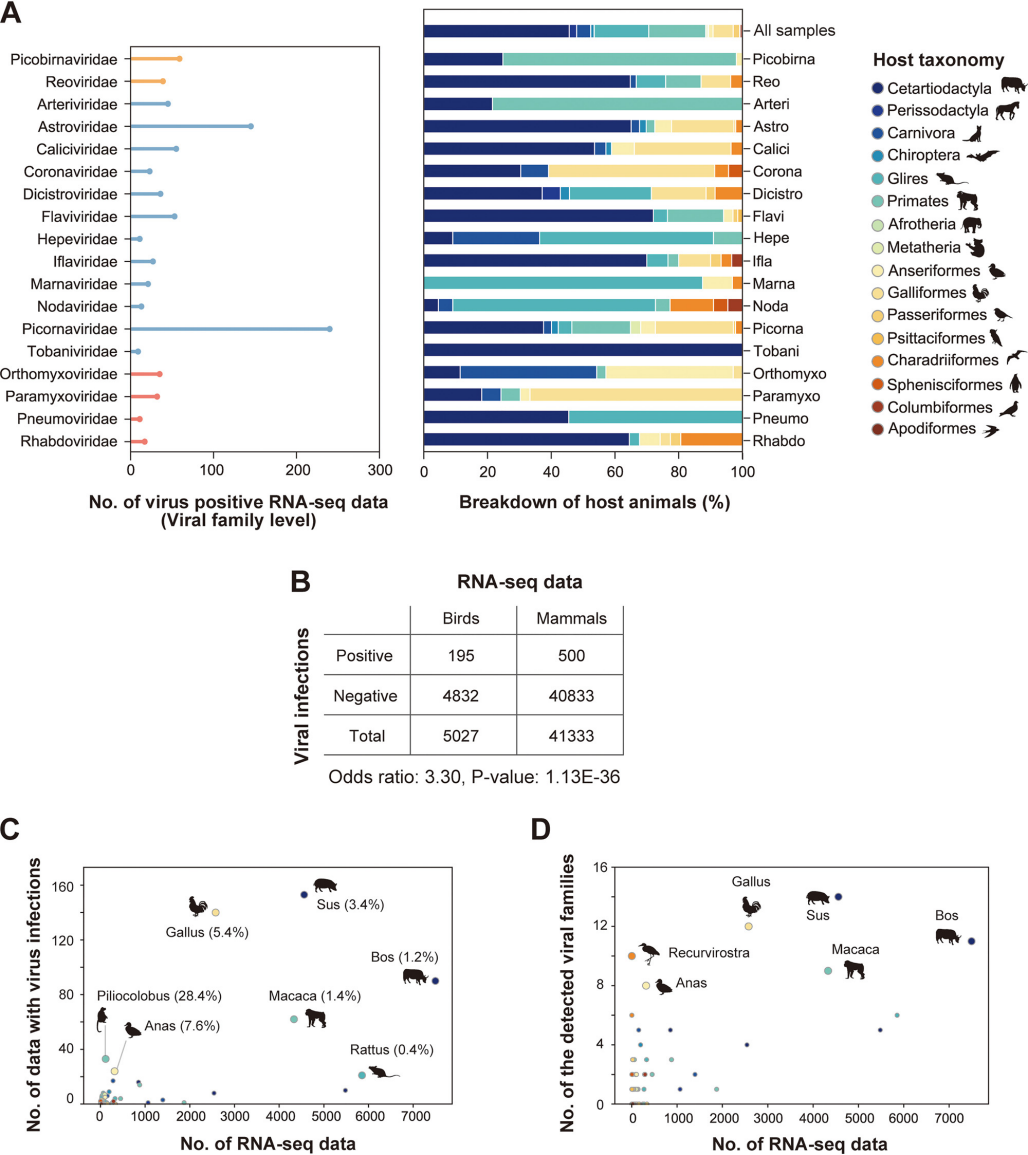
RNA viral infections in the public sequencing data. (A) RNA viral infections detected in public sequencing data. Left panel, the *x* axis indicates the number of virus-positive RNA-seq data, and the *y* axis indicates viral families. Although infections by 22 RNA viral families were identified in this study, 18 families that were detected in more than five RNA-seq data are shown here. Bar colors correspond to the Baltimore classification: orange, dsRNA viruses; blue, ssRNA(+) viruses; red, ssRNA(−) viruses. Right panel, breakdown by host animals in which viral family infections were detected. The filled colors correspond to the host taxonomy shown in the key. The top row indicates the animal-wide breakdown of all RNA-seq data used in this study. (B) Comparison of viral detection rates between avian and mammalian samples. The table shows the numbers of RNA-seq data with and without viral infections. The odds ratio and *P* value were obtained by Fisher’s exact test. (C) Scatterplot between the numbers of RNA-seq data investigated in this study (*x* axis) and those with viral infections (*y* axis). Each dot indicates the animal genus. Dot colors correspond to the host taxonomy shown in panel A. The animal genera in which viral infections were detected in ≥20 samples are annotated with the representative animal species silhouettes. The percentages in parentheses indicate the ratios of virus-positive RNA-seq data to the investigated data. (D) Scatterplot between the number of RNA-seq data investigated in this study (*x* axis) and those of detected viral families (*y* axis). Each dot indicates the animal genus. Dot colors correspond to the host taxonomy shown in panel A. The animal genera in which eight or more viral families were detected are annotated with the representative animal species silhouettes.

More than 46,000 mammalian and avian RNA-seq data were used to investigate infections by 33 RNA virus families reported to infect vertebrates. Consequently, we identified 882 infections of 22 RNA virus families in 695 sequencing data from 53 host species (Fig. 2A). These results indicate that analyzing public sequencing data by metagenomic analysis is useful for identifying hidden viral infections.

### Frequent detection of diverse virus families in bird samples.

Many viral infectious diseases associated with birds have been reported so far (12), such as influenza A virus (13, 14) and West Nile virus (15). In this study, we frequently detected viral infections in bird samples (Fig. 2B). The odds ratio of RNA virus detection in birds to that in mammals was 3.3. Furthermore, among the investigated species, we found relatively high viral detection rates in *Gallus* and *Anas* species, at 5.4% and 7.6%, respectively (Fig. 2C). We also found infections of 12 and 8 virus families in *Gallus* and *Anas* species, respectively (Fig. 2D). These results indicate that birds, especially *Gallus* and *Anas* species, are frequently infected with various virus families, suggesting that these species are reservoirs for a wide variety of viruses (see Discussion).

### Identification of unknown reservoir hosts at virus family levels.

**FIG 3:**
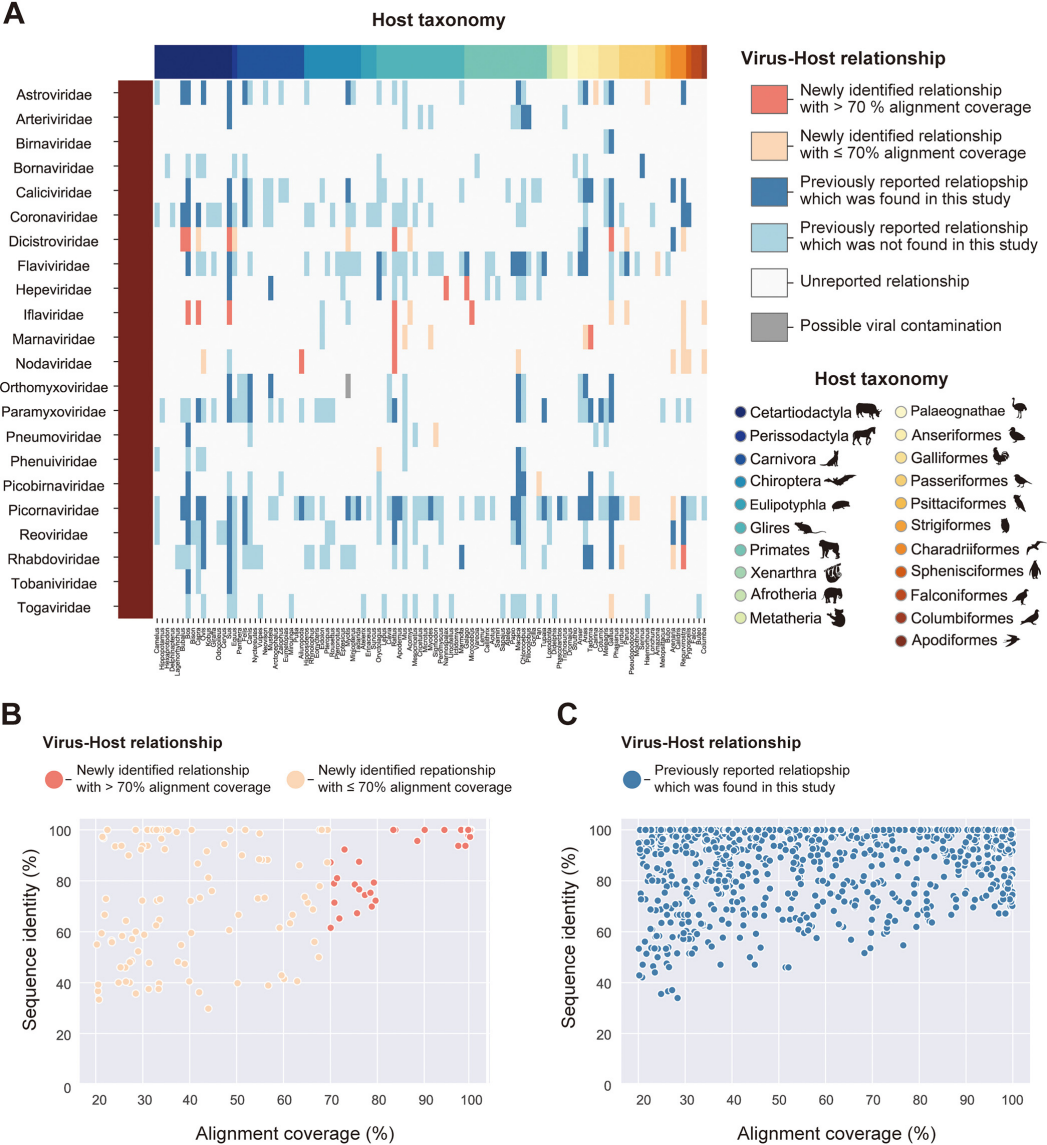
Search for unknown reservoir hosts and novel virus sequences. (A) Heatmap showing the newness of virus-host relationships. Rows indicate viral families that reportedly infect vertebrate hosts. Columns indicate animal genera, and filled colors correspond to the host taxonomy shown in the lower right corner. Heatmap colors are according to six categories of virus-host relationships shown in the upper right corner: a relationship was newly identified in this study and a viral infection was detected with  >70% alignment coverage (coral), a relationship was newly identified in this study but the viral infection was detected with  ≤70% alignment coverage (salmon), a relationship was previously reported and the viral infection was also detected in this study (blue), a relationship was previously reported but the viral infection was not detected in this study (light blue), a relationship was unreported so far (white), and a relationship was newly identified in this study but it may be attributed to contamination (gray) (see Discussion). (B and C) Scatterplot between alignment coverages (*x* axis) and sequence identities with known viruses (*y* axis). Each dot represents the viral infections identified in this study. Viral infections related to novel virus-host relationships are shown in panel B, and those related to known relationships are shown in panel C. The dot colors correspond to the virus-host relationships shown in panel A. Sequence identity represents the maximum value of the percentage of identical matches obtained by TBLASTX.

To identify novel virus-host relationships at virus family levels, we compared our data with known virus-host relationships provided in the Virus-Host Database (Virus-Host DB) (16) (Fig. 3A; Data Set S4). This database lists virus-host relationships based on the identification of viral sequences from a host animal. We found 50 newly identified virus-host relationships using this database for comparison, and 17 of them were identified with more than 70% alignment coverage. Notably, we identified nearly complete genomic sequences classified into the family *Hepeviridae* in *Spalax* and *Galago* species for the first time. These discoveries expanded our understanding of hepeviral host ranges (details of the viral characteristics are described in “Hepeviruses in blind mole-rats and a galago: expanding understanding of the hepatitis E virus host range”). A novel relationship was also identified between the family *Rhabdoviridae* and *Recurvirostra* species. We did not perform further investigations because the complete rhabdovirus genome could not be obtained, although the alignment coverage was more than 70%. Additionally, novel virus-host relationships were also found in the families *Dicistroviridae*, *Iflaviridae*, *Marnaviridae*, and *Nodaviridae*, suggesting that these viral host ranges are broader than previously expected. It should be noted that these relationships might be due to contamination with environmental viruses in feces or foodstuffs, because few species in these viral families have been reported to infect mammals or birds (17–20) (see Discussion).

10.1128/mBio.01638-21.6 DATA SET S4 Information on manual curation for virus-host relationships. Download Data Set S4, XLSX file, 0.7 MB. Copyright © 2021 Kawasaki et al. 2021 Kawasaki et al. https://creativecommons.org/licenses/by/4.0/ This content is distributed under the terms of the Creative Commons Attribution 4.0 International license.

### Investigation of novel viruses with complete genomic sequences.

**FIG 4:**
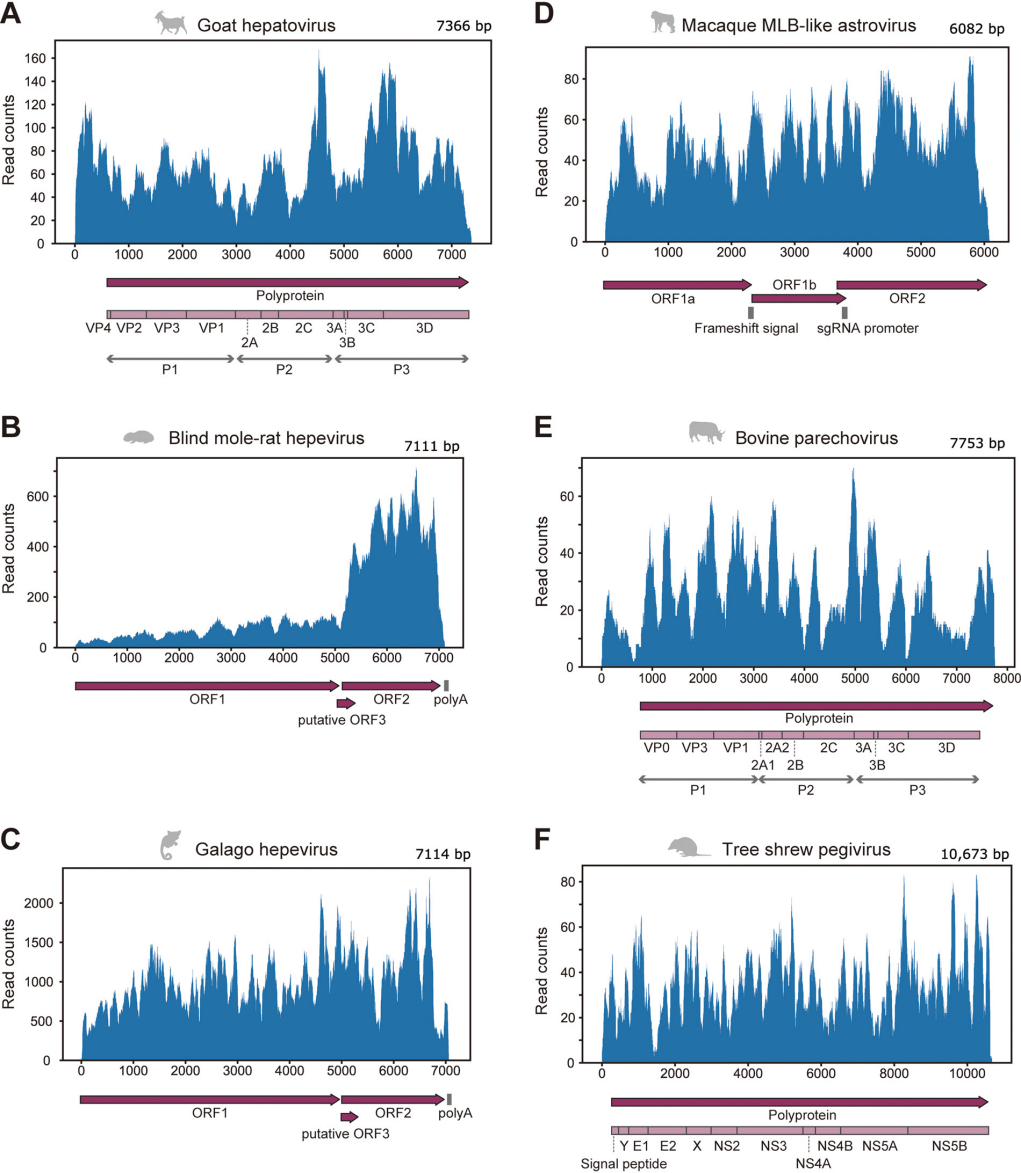
Mapping analysis using RNA-seq data in which the full-length viral genome was identified. (A to F) Read distributions were mapped to the genomic sequence of goat hepatovirus (A), blind mole-rat hepevirus (B), galago hepevirus (C), macaque MLB-like astrovirus (D), bovine parechovirus (E), and tree shrew pegivirus (F). The upper panel shows the virus genomic positions (*x* axis) and read counts at each position (*y* axis). The lower panel shows genomic annotations, such as protein-coding regions or signal sequences. Dark purple arrows indicate open reading frames (ORFs) in the viral genome. Light purple boxes show mature proteins predicted based on aligned positions with reference viruses (see Materials and Methods). Gray vertical lines indicate nucleotide sequence features, such as polyadenylation signal [poly(A)], ribosomal frameshift signal (frameshift signal), and promoter sequence for subgenomic RNA synthesis (sgRNA promoter).

**FIG 5:**
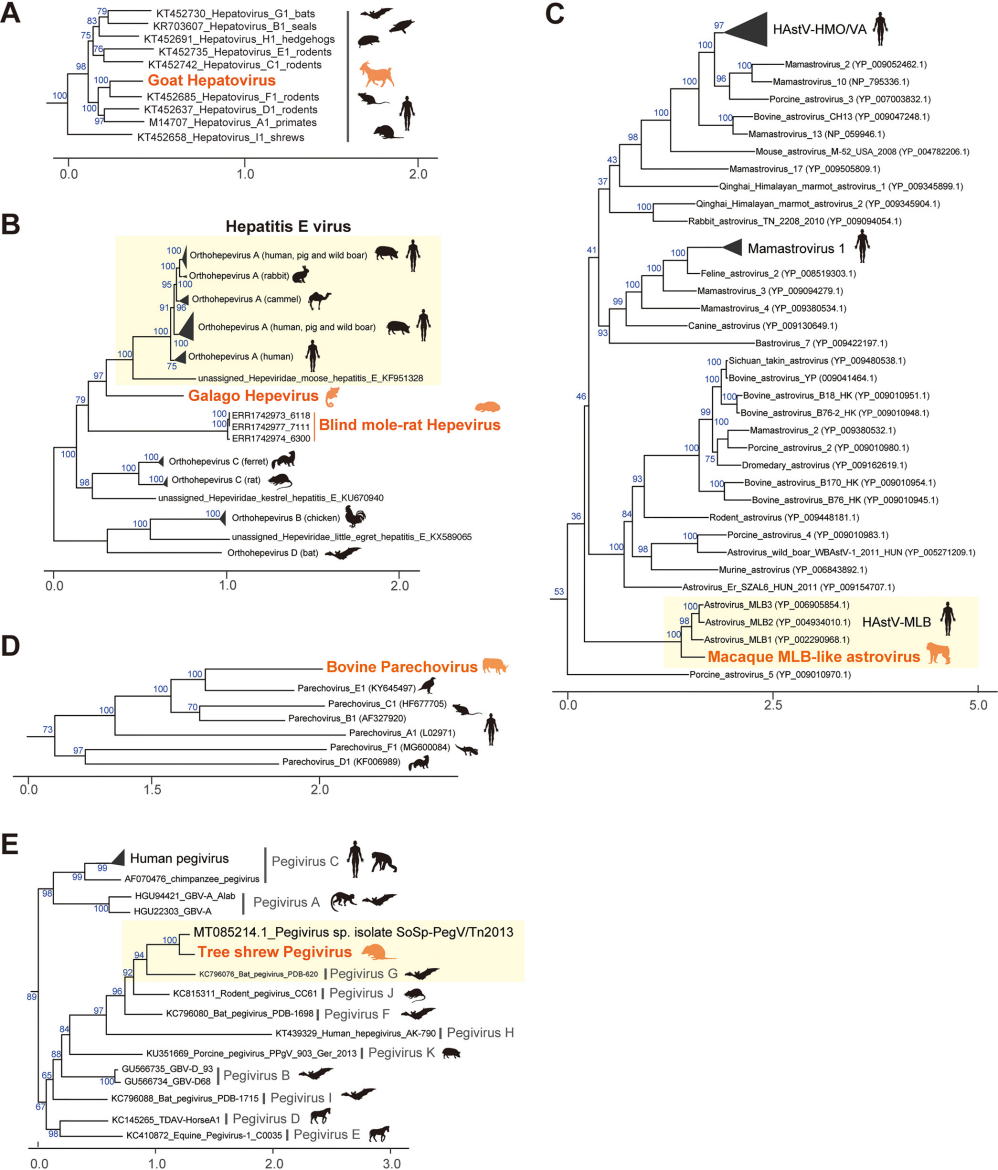
Characterization of virus sequences identified in this study. (A to E) Phylogenetic analyses of the genus *Hepatovirus* of the family *Picornaviridae* (A), the family *Hepeviridae* (B), the genus *Mamastrovirus* of the family *Astroviridae* (C), the genus *Parechovirus* of the family *Picornaviridae* (D), and the genus *Pegivirus* of the family *Flaviviridae* (E). These phylogenetic trees were constructed based on the maximum likelihood method (see Materials and Methods). Orange labels indicate viruses identified in this study, and colored animal silhouettes indicate the viral host species. Black labels and animal silhouettes indicate known viruses and their representative hosts, respectively. Scale bars indicate the genetic distance (substitutions per site). Blue numbers on branches indicate the bootstrap supporting values (%) with 1,000 replicates. Yellow boxes highlight viruses genetically similar to the novel virus identified in this study.

**FIG 6:**
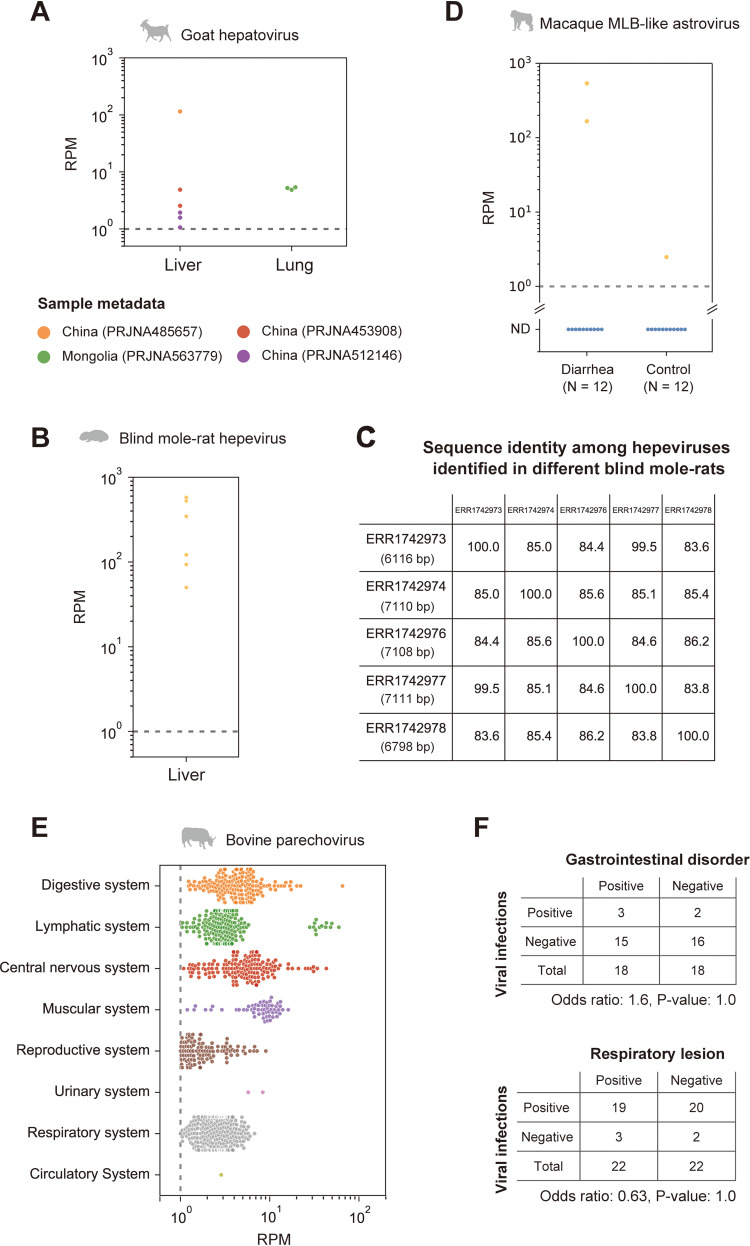
Detection of viral infections in the natural host population. (A, B, and E) Investigation of viral infections in the natural host population by quantifying viral reads for goat hepatovirus (A), blind mole-rat hepevirus (B), and bovine parechovirus (E). The graph indicates the viral read amount (read per million reads [RPM]) in each tissue or organ system. The gray dotted line indicates the criterion used to determine viral infections (RPM, 1.0). The lower portion of panel A shows the sample metadata. (C) Comparison of nucleotide sequence identities among the hepeviral sequences identified in five different blind mole-rats. The numbers in parentheses in each row are the total number of aligned sites between the viral contigs identified in each individual and the blind mole-rat hepevirus identified in accession no. ERR1742977. (D) Quantification of the macaque MLB-like viral infection levels in the patients with diarrhea and control macaque monkeys. The *x* axis indicates the diagnosis for the 24 monkeys, and the *y* axis indicates the RPM. The average RPM for each individual is plotted because six samples were collected from each individual. The dotted line indicates the criterion used for detecting viral infections (RPM, 1.0). We considered samples with RPMs below the criterion as nondetectable (ND). (F) Association between the parechovirus infections and symptoms. The tables show the number of RNA-seq data with and without parechovirus infections in two independent studies, which provide diagnostic information for gastrointestinal disorder (upper panel) and respiratory lesion (lower panel). The odds ratios and *P* values were obtained by Fisher’s exact test.

To identify novel sequences comparable to a complete viral genome, we simultaneously analyzed sequence identity with known viruses and alignment coverages with reference viral genomes (Fig. 3B and C). We found some viral sequences showing low sequence identity with known viruses and high alignment coverage, which were expected to be novel viruses with a nearly complete genome. Therefore, we further characterized these viral sequences by phylogenetic analyses, annotations of viral genomic features, and quantification of viral reads in RNA-seq data (Fig. 4 to 6; Fig. S2 and Data Sets S6 and S7). Consequently, we discovered six viruses with high read coverages over the entire genome (Fig. 4): hepatovirus in a goat, hepeviruses in blind mole-rats and a galago, astrovirus in macaque monkeys, parechovirus in a cow, and pegivirus in tree shrews.

10.1128/mBio.01638-21.2 FIG S2 Comparison with the ICTV species demarcation criteria. (A to C) Genetic distances among the amino acid sequences of novel and known viruses in the genera *Hepatovirus* (A), *Parechovirus* (B), and *Pegivirus* (C). The *x* axis indicates the proportion of different sites: p-distance. Each dot shows the amino acid sequence p-distance between the novel and known virus species. The ICTV species demarcation criteria are shown as orange dotted lines: greater than 0.3 in polyprotein, P1, and 2C + 3CD regions for hepatoviruses (A), greater than 0.3 in polyprotein, P1 regions, and 0.2 in 2C + 3CD region for parechoviruses (B), and greater than 0.31 in the NS3 region and 0.31 to 0.36 in the NS5B region for pegiviruses (C). Download FIG S2, PDF file, 0.1 MB. Copyright © 2021 Kawasaki et al. 2021 Kawasaki et al. https://creativecommons.org/licenses/by/4.0/ This content is distributed under the terms of the Creative Commons Attribution 4.0 International license.

### Goat hepatovirus: the first report on hepatoviral infections in livestock animals.

Hepatitis A virus (HAV), belonging to the genus *Hepatovirus* of the family *Picornaviridae*, can cause acute and fulminant hepatitis and is typically transmitted via fecal-oral routes, including contaminated water or foods (21). The World Health Organization (WHO) reported that HAV infections resulted in death in over 7,000 people in 2016 (https://www.who.int/news-room/fact-sheets/detail/hepatitis-a). Here, we identified a hepatoviral infection in a goat sample (Fig. 4A and 5A). To our knowledge, this is the first report of hepatoviral infection in livestock animals.

We further analyzed hepatovirus prevalence in a natural host population by quantifying the viral reads in other goat RNA-seq data, because this virus was initially identified in only one goat sample. Among 1,593 samples, we found the viral infections in nine samples from four independent studies with >1.0 read per million reads (RPM) (Fig. 6A; Data Set S7). The goat hepatoviral infections were detected in liver and lung samples, suggesting that goat hepatovirus can infect tissues other than the liver. Although the lungs are not considered preferential tissues for hepatoviral replication, a previous report also detected hepatoviral RNAs in the lungs of seals (22). We also should note that the presence of goat hepatoviral RNA in the lung samples may be due to viremia. The infected goat samples were collected in East Asia, including China and Mongolia. Therefore, goat hepatoviruses may be prevalent in the natural host population, suggesting that this virus can be a new threat to public health through the contamination of water and foods by infected animals.

10.1128/mBio.01638-21.9 DATA SET S7 Sample metadata in which the six novel viral infections were detected. Download Data Set S7, XLSX file, 0.09 MB. Copyright © 2021 Kawasaki et al. 2021 Kawasaki et al. https://creativecommons.org/licenses/by/4.0/ This content is distributed under the terms of the Creative Commons Attribution 4.0 International license.

### Hepeviruses in blind mole-rats and a galago: expanding understanding of the hepatitis E virus host range.

Several million infections of hepatitis E virus (HEV) are estimated to occur worldwide; the WHO reported approximately 44,000 deaths due to HEV infection in 2015 (https://www.who.int/news-room/fact-sheets/detail/hepatitis-e). Here, we found hepeviruses, classified into the same viral family as HEV, in blind mole-rats and a galago for the first time (Fig. 3A and 4B and C). Phylogenetic analysis indicated that these hepeviruses formed a single cluster with moose HEV (23) and members of orthohepevirus A that infect humans, pigs, rabbits, and camels (24) (Fig. 5B). However, the hepeviruses identified in this study appeared to have an early divergence from the HEV common ancestor. These results suggest a high diversity and broader host range of HEV-like viruses.

The blind mole-rat hepevirus was identified in the host livers, which coincides with the tissue tropism of HEV (25). Additionally, we found that the 3′ portion of the blind mole-rat hepevirus genome was highly transcribed (Fig. 4B), suggesting the transcription of subgenomic RNAs (26). In contrast, we could not determine the tissues infected by the galago hepevirus, because the relevant metadata were not available. Furthermore, we did not observe a clear read-mapping pattern that suggests any subgenomic RNA transcription in the galago sample (Fig. 4C).

We also investigated the spread of these hepeviruses in a natural population using RNA-seq data from blind mole-rats and galagos. Among 91 RNA-seq data from blind mole-rats, we detected hepeviral infections in six samples (Fig. 6B). These infected individuals were captured and kept as laboratory animals in Israel by the same researchers (Data Set S7). There were two possibilities about when the hepeviruses had infected blind mole-rats: the hepeviruses had already infected these blind mole-rats when they were captured, or the viral infections had spread during the maintenance of these individuals in the laboratory. To explore these possibilities, we investigated the interindividual diversity of the hepevirus sequences and found that these blind mole-rats were infected with relatively diverse hepeviruses representing nucleotide sequence identities ranging from 83.6% to 99.5% (Fig. 6C). These results suggest that several individuals had already been infected with distinct hepeviruses in the wild before being captured. The galago hepeviral infections were detected in only two samples originating from a study in which we first identified the virus (Data Set S7). This may be simply because only four galago RNA-seq data obtained from the same study were available.

### MLB-like astrovirus detected in macaque monkeys with chronic diarrhea.

We found astroviruses that were genetically similar to human astrovirus MLB (HAstV-MLB) in fecal samples of macaque monkeys (Fig. 5C). Although HAstV-MLB infections are typically asymptomatic (27, 28), several studies have reported detection of this virus in cases with diarrhea (29), encephalitis (30), or meningitis (31). Interestingly, the macaque MLB-like astrovirus was found in macaque monkeys with chronic diarrhea. We analyzed the viral read amounts in the patient (*n* = 12) and control (*n* = 12) monkeys to assess the association between MLB-like astroviral infections and symptom prevalence (Fig. 6D; Data Set S7). Abundant MLB-like astroviral reads were detected in two patients, suggesting that the viral infections are associated with host symptoms. However, we did not observe the viral infection in other patients; furthermore, we found the infection in a control individual, although the viral read amount was approximately 100 times less than that of the patients. Additionally, a previous study reported that monkeys in which partial sequences of MLB-like astroviruses were detected had no obvious clinical signs, including diarrhea (32). Thus, further experiments are needed to clarify the pathogenesis of MLB-like astrovirus. Considering that there is no current experimental system for examining HAstV-MLB infections (28), our findings suggest that macaque monkeys can be used as animal model systems for researching MLB-like astroviruses.

### Silent infections of bovine parechovirus having a broad tissue tropism.

Human parechovirus infection is especially problematic in infants and young children. Although most parechovirus infections are considered asymptomatic, their infections have been reported in patients with respiratory, digestive, and central nervous system disorders (33). In this study, we identified a parechovirus, classified into the family *Picornaviridae*, in the lower digestive tract of a cow (Fig. 5D). Despite the broad host range of parechovirus, including mammals, birds, and reptiles (34), to our knowledge, this is the first report on parechovirus infections in livestock animals.

Phylogenetic analysis indicated that this parechovirus was closely related to the falcon parechovirus, a member of parechovirus E. Next, we compared the bovine parechovirus with the ICTV species demarcation criteria (34) to investigate whether this virus is a novel species (Fig. S2B). Consequently, we found that the bovine parechovirus was distant enough from other known parechovirus species and could be considered a separate species based on the following criteria: divergence of amino acid sequences in polyprotein (37.8%), P1 protein (37.8%), and 2C + 3CD protein (29.9%). Therefore, we propose that this virus belongs to a new species in the genus *Parechovirus*.

We also investigated the prevalence of this parechovirus infection in a natural host population using public RNA-seq data of cows (Fig. 6E; Data Set S7). Among 8,284 samples, we detected the parechovirus infections in 944 samples from eight independent studies with >1.0 RPM. The viral infections were detected in various tissues, such as tissues from the digestive, lymphatic, and central nervous systems. These results suggest a broad tissue tropism of the bovine parechovirus. To assess the parechovirus pathogenicity, we analyzed the viral prevalence among 36 or 44 samples with a diagnosis for a gastrointestinal disorder or respiratory lesion, respectively. We did not observe a significant association between the viral infections and the presence/absence of symptoms in these two studies (Fig. 6F). These results indicate that bovine parechovirus infections may be asymptomatic, similar to the typical outcome of human parechoviral infections. Furthermore, this also suggests that infected cows can spread parechoviral infections as silent reservoirs.

### Geographical expansion of tree shrew pegivirus infection associated with host migration.

We found a pegivirus belonging to the genus *Pegivirus* of the family *Flaviviridae* in tree shrew liver samples. Phylogenetic analysis indicated that this pegivirus was closely related to the pegivirus G identified in various bat species (Fig. 5E). According to the ICTV species demarcation criteria (35), this virus appeared to be the same species as pegivirus G, because the amino acid sequence identity in the NS5B gene was 70.9% (Fig. S2C). These results indicate that pegivirus G can infect distinct host lineages: tree shrews and bats.

We also investigated the pegiviral infections in other tree shrew samples by mapping analysis. Among the 59 samples, the pegiviral infections were detected in four samples collected from a research colony in the United Kingdom (Data Set S7). A recent report partially identified a pegiviral sequence (GenBank accession no. MT085214.1) in tree shrews collected in Southeast Asia (36) which showed 84.9% nucleotide sequence identity to the pegivirus identified in this study (Fig. 5E). These results indicate that tree shrew pegivirus infections were found in Asia and Europe, suggesting an expanding geographic distribution of pegivirus G along with host animal transportation as experimental resources. Thus, the global trade of host animals may lead to the spread of pegiviral infections hidden in tree shrews.

## DISCUSSION

Metagenomic analysis is a powerful approach for surveying viral infections (4, 5). Extensive deep-sequencing data have been accumulated in public databases, which can be used for identifying viral infections. In this study, we analyzed the publicly available RNA-seq data to search for hidden RNA viral infections in mammalian and avian species and subsequently identified approximately 900 infections by 22 RNA virus families (Fig. 1 and 2). These results indicate that reusing public sequencing data is a cost-effective approach for identifying viral infections. Furthermore, we discovered six novel viral genomes in livestock, wild, and experimental animals (Fig. 4 and 5). Some of these viruses were detected in different individuals, suggesting that the viral infections may have already spread in the natural host population (Fig. 6). Overall, our work demonstrates the reusability of public sequencing data for surveying infections by both known and unknown viruses.

In this study, we determined viral infections by a combination of sequence assembly and the alignment coverage-based method to solve several issues in viral metagenomic analysis (Fig. 1A). One of the problems is detecting infections in data with a small number of viral reads, because almost all public sequencing data were collected without using virus enrichment strategies. The result that most virus contigs were shorter than the reference viral genomes reflects this difficulty (Fig. 1B and C). To resolve this issue, we determined viral infections by the alignment coverage-based method, which uses relatively short viral sequences as clues (Fig. 1A; see Fig. S1 in the supplemental material). Consequently, we succeeded in detecting approximately 900 RNA viral infections in public sequencing data (Fig. 2A). Another problem in viral metagenomic analysis is the viral detectability depending on sequence similarity with known viruses. We here discovered six nearly complete viral genomes (Fig. 4 and 5) by sequence assembly and BLAST screening (Fig. 1A). Notably, these viral infections were undetectable in almost all samples, even at the virus family and genus levels, by the NCBI SRA Taxonomy Analysis Tool (https://github.com/ncbi/ngs-tools/tree/tax/tools/tax), which determines the taxonomic composition of reads in RNA-seq data without sequence assembly (Data Set S7). These results indicate that our method can identify novel viruses with full-length genomes, which would effectively elucidate virus diversity. Taken together, our strategy using sequence assembly and the alignment coverage-based method can efficiently detect known and unknown viral infections in publicly available sequencing data.

However, there are still several challenges for identifying viral infections in public sequencing data. First, we could not determine complete viral sequences mostly (Fig. 3B and C). Further improvements in sequence assembly efficiency (37) or integrative analysis using short- and long-read sequence datasets (38) can solve this problem. Second, there may be a bias in virus detection using public sequencing data depending on the genomic types of the viruses. Among the 882 viral infections identified in this study, 77.0% were positive-sense single-stranded RNA [ssRNA(+)] viral infections, whereas 11.5% and 11.5% were double-stranded RNA and negative-sense single-stranded RNA viral infections, respectively (Fig. 2A). The RNA-seq procedure, such as enrichment of polyadenylated [poly(A)] transcripts, can be relevant to this bias because many ssRNA(+) viruses have a poly(A) tract at the 3′ end of their genome (39). Alternatively, this bias may result from a repertoire of reference viral genomes used for the viral search (Fig. 1C), which can be solved in the future by database expansion. Third, our method demands relatively abundant computational resources, including operation time, for determining viral infections in each of the RNA-seq data. We reconstructed viral sequences from RNA-seq data according to several steps: mapping analysis for excluding host transcripts, *de novo* sequence assembly using unmapped reads, and BLASTX screening for identifying viral sequences (Fig. 1A). In contrast, another study performed a search for viral RNA-dependent RNA polymerase proteins in translated nucleotide sequences, which enabled the authors to screen for viral infections in approximately 5.7 million public sequencing data within 11 days (11). Considering that the number of public sequencing data will continue to increase, platform development and maintenance, which can save computational resources, are necessary for continuing such viral surveillance.

Another challenge in viral metagenomic analysis using public data is distinguishing true viral infections from contamination. The reuse of public sequencing data requires careful consideration to determine viral infections, since it is difficult to control the effects of contamination at the sampling and sequencing steps. To address this issue, we performed integrative analyses using sample metadata and sequence information, including sequence similarity and alignment coverage with known viruses (see Materials and Methods). Consequently, we found several possible contamination cases: influenza A virus in a *Myotis* bat, vesicular stomatitis Indiana virus (VSV) in cultured chicken cells, mammalian orthorubulavirus 5 (parainfluenza virus 5 [PIV5]) in cultured cells and quail egg samples, and Kadipiro virus (KDV) in rat samples (Fig. 3A; Data Set S3). For example, the influenza A viral nucleotide sequence identified in a bat sample showed 100% similarity to a laboratory strain of influenza A virus [A/WSN/1933(H1N1)]. Considering that the bat sample was collected in 2012, it is difficult to expect that such a highly similar influenza A virus was maintained for approximately 80 years. Likewise, the infections of VSVs and PIVs were also identified with approximately 100% sequence similarity to the reference viral sequences (Data Set S3). VSV is frequently used as an experimental tool, for example, as a pseudotype virus (40). Previous studies have also reported possible contamination of PIV5 in cultured cells (41, 42). Additionally, it has been reported that KDV RNA might be a contaminant in the nucleic acid extraction kit (43). Therefore, we excluded these viral infections to avoid counting false positives. These cases emphasize the importance of multilayered validations for viral infections found by viral metagenomic analysis alone.

10.1128/mBio.01638-21.5 DATA SET S3 Information on possible viral contamination excluded from the totalization. Download Data Set S3, XLSX file, 0.01 MB. Copyright © 2021 Kawasaki et al. 2021 Kawasaki et al. https://creativecommons.org/licenses/by/4.0/ This content is distributed under the terms of the Creative Commons Attribution 4.0 International license.

Further research efforts to elucidate viral diversity are necessary to prepare for a possible future viral pandemic (1, 5). A strategic approach, such as determining the host samples used for a virus search based on the expectation of viral infection frequency or viral diversity, is necessary. It has been discussed that birds may be high-risk viral hosts of zoonoses because of their high species diversity and wide habitat range (12). In this study, we found that viral infections were more frequently detected in birds, especially *Gallus* and *Anas* species (Fig. 2B to D). Furthermore, among 217 viral infections identified in *Gallus* and *Anas* samples, 78 infections (35.9%) showed less than 95% amino acid sequence similarity with known viruses, suggesting that these sequences may be derived from unknown viruses. Therefore, further viral metagenomic analyses targeting bird samples may effectively detect viral infections, including unknown ones.

In conclusion, we demonstrated the reusability of public sequencing data for monitoring viral infections and discovering novel viral sequences and elucidated diverse RNA viruses hidden in animal samples. Further virological analyses, such as virus isolation, immunohistochemistry, and epidemiological surveys, are warranted to understand virus-host relationships, infectivity, and pathogenicity. Our findings also emphasize the necessity of continuous surveillance for viral infections by using public sequencing data to prepare for future viral pandemics, as well as the importance of developing a fundamental bioinformatics platform for surveillance (11, 44).

## MATERIALS AND METHODS

### Sequence assembly using publicly available RNA-seq data.

We collected RNA-seq data of 41,332 mammals (169 genera and 228 species) and 5,027 birds (70 genera and 83 species) from the NCBI Sequence Read Archive (SRA) database (8) during June and July 2019 according to the following search conditions: {(“Mammalia”[Organism] OR “Mammals”[All Fields]) AND (“biomol rna”[Properties] AND “library layout paired”[Properties] AND “filetype fastq”[Properties]) NOT (“Homo sapiens”[Organism]) NOT (“Mus musculus”[Organism])] and [(“Aves”[Organism] OR “Aves”[All Fields]) AND (“biomol rna”[Properties] AND “library layout paired”[Properties] AND “filetype fastq”[Properties])}. The RNA-seq data were downloaded from the NCBI SRA database by pfastq-dump (https://github.com/inutano/pfastq-dump) and preprocessed using fastp (version 0.20.0) (45) with options “-l 35,” “-y -3,” “-W 3,” “-M 15,” and “-x”.

Sequence assembly was conducted by (i) mapping reads to the host or sister species genome and (ii) *de novo* assembly of sequences using unmapped reads. First, we performed a mapping analysis to exclude the reads originating from host transcripts and endogenous viral elements. We mapped the reads in each of the RNA-seq data to the host genome by HISAT2 (version 2.1.0) (46) with the default parameters or used the sister species genomes of the host in the same genus when the host genome data were not available. Unmapped reads were extracted by Samtools (version 1.9) (47) and Picard (version 2.20.4) (http://broadinstitute.github.io/picard). When the relevant genome data were unavailable, the preprocessed reads were directly used for sequence assembly. Sequence assembly was conducted by SPAdes (version 3.13.0) (48) or metaSPAdes (version 3.13.0) (49) with *k*-mers of 21, 33, 55, 77, and 99. Finally, we excluded contigs with lengths shorter than 500 bp by Seqkit (version 0.9.0) (50) and then clustered the contigs showing 95.0% nucleotide sequence similarity by cd-hit-est (version 4.8.1) (51). Consequently, we obtained 422,615,819 contigs and used them for subsequent analyses. We list the SRA run accession numbers, genome files used for mapping analysis, and sequence assembly tools in Data Set S1 in the supplemental material.

### Identification of contigs originating from RNA viruses.

To determine the origins of the contigs, we analyzed the sequence similarity between the contigs and known sequences in BLASTX screening (version 2.9.0) (52). First, we performed BLASTX searches with the options “-word_size 2,” “-evalue 1E-3,” and “max_target_seqs 1” using a custom database consisting of RNA viral proteins. We constructed the custom database by downloading the viral protein sequences of the realm *Riboviria* from NCBI GenBank (version 20190102) (53) and clustering the sequences showing 98.0% similarity by cd-hit (version 4.8.1). Second, to confirm that the contigs are not derived from organisms other than viruses, we further performed BLASTX searches with the options “-word_size 2,” “-evalue 1E-4,” and “-max_target_seqs 10” using the NCBI nr database (versions 20190825 to 20190909 were used for screening contigs in mammalian data, and versions 20190330 to 20190403 were used for screening contigs in avian data). We determined the contig origins by comparing the bitscores in the first and second BLASTX screenings. Consequently, we obtained 17,060 contigs that were deduced to encode RNA viral proteins.

### Total of RNA viral infections in public RNA-seq data.

Since most viral contigs were shorter than the reference viral genomes (Fig. 1B and C), we sought to determine viral infections based on the alignment coverage-based method (Fig. 1A). First, we performed sequence alignment by TBLASTX (version 2.9.0) using viral contigs from the RNA-seq data and complete viral genomes in the NCBI RefSeq genomic viral database (version 20200824). Next, we calculated the alignment coverage with the genome of each viral species, i.e., the proportion of aligned sites in the entire reference viral genome. In this study, we considered that an infection of the viral family was present if the alignment coverage was greater than 20%. Validation of this totalization method and evaluation of the criteria are described in the next section (Fig. S1). Furthermore, we manually checked sequences with more than 70% alignment coverage and more than 95% identity with known viruses in the TBLASTX alignment to examine possible contamination with laboratory viral strains, as well as experimentally inoculated viruses. We excluded experimentally inoculated viral infections (Data Set S2) and possible contamination (Data Set S3) from the final totals (Fig. 2A).

10.1128/mBio.01638-21.4 DATA SET S2 Information on RNA-seq data from experimental infection with viruses. Download Data Set S2, XLSX file, 0.2 MB. Copyright © 2021 Kawasaki et al. 2021 Kawasaki et al. https://creativecommons.org/licenses/by/4.0/ This content is distributed under the terms of the Creative Commons Attribution 4.0 International license.

### Validation of the procedure used to total viral infections.

Using samples obtained from viral infection experiments, we first compared the alignment coverage-based method with that based on viral read amounts in order to validate the detection rate of viral infections of our method (Fig. S1; Data Set S2). We obtained the read amounts derived from experimentally infected viruses from the NCBI SRA Taxonomy Analysis Tool results (https://github.com/ncbi/ngs-tools/tree/tax/tools/tax). The calculation procedure for alignment coverage between viral contigs in each of the RNA-seq data and viral reference genomes is described in the previous section. We observed a positive correlation between the alignment coverage and viral read amounts (Pearson’s correlation coefficient, 0.19; *P* value, 1.87E-6) (Fig. S1A). Among the samples collected from experiments of viral infections, the true-positive rate (the detection rate of experimentally inoculated viruses) was 88.3% and the false-positive rate (the rate that mock samples were determined to be infected samples) was 62.5% when we used 20% alignment coverage as the criterion for determining viral infections (Fig. S1B). The relatively high false-positive rate may be due to similar amounts of viral reads in some mock samples and in infected samples (Fig. S1A). Next, we analyzed the association between alignment coverages and viral genome size (Fig. S1C) because the detectability of viral infections in our method may depend on the reference viral genome size. As expected, we observed a tendency for viruses with small genomes to be detected with relatively high alignment coverage. However, more than 80% of experimentally infected viral infections were detected with more than 20% alignment coverage, regardless of the viral genome size. Based on these results, we established the alignment coverage of 20% to total the viral infections. Consequently, we identified a total of 1,410 RNA viral infections, including 503 infections in samples from viral infectious experiments (Fig. S1D).

### Collection of information on experimentally infected viruses.

To exclude experimentally infected viruses from the final totals, we investigated the experimental background of RNA-seq data. We first collected the experimental descriptions of RNA-seq data, including the title and abstract from the NCBI BioProject database (54). Then, we manually checked the terms relevant to viral infections in the descriptions, focusing on viral name abbreviations and viral vector usage. We list the obtained information about viral infection experiments in Data Set S2.

### Summary of virus-host relationships.

To identify novel reservoir hosts at the viral family levels, we compared the virus-host relationships identified in this study with the data set provided by the Virus-Host DB (version 20200629) (16). We defined a “novel virus-host relationship” as one in which the viral sequence has not been reported in the host. The virus-host relationships at the viral family level were categorized as (i) a novel relationship detected with >70% alignment coverage, (ii) a novel relationship detected with ≤70% alignment coverage, (iii) a known relationship that was also detected in this study, (iv) a known relationship that was not identified in this study, (v) a relationship unreported so far, and (vi) a novel relationship which was possibly derived from contamination (see Discussion). To avoid misclassification of the relationships, we analyzed reports manually by searching the NCBI PubMed and Nucleotide databases using a combination of the host genus and viral family names: for example, [“Pan” AND “Picobirnaviridae”]. The results of the manual curation are listed in Data Set S4.

### Characterization of viral genomic architectures.

Open reading frames (ORFs) and polyadenylation signals in the viral genomes were predicted by the SnapGene software (snapgene.com). The positions of mature proteins, frameshift signal sequences, and subgenomic RNA promoter sequences were predicted based on sequence alignment using novel and reference viral sequences. The sequence alignments were constructed by MAFFT (version 7.407) (55) with the option “–auto”. The reference viral sequences used for the genome annotations are listed in Data Set S5. The viral sequences identified in this study are registered under the following accession numbers: BR001715 to BR001732 and BR001751.

10.1128/mBio.01638-21.7 DATA SET S5 Accession numbers of viral sequences used for phylogenetic analyses, viral genomic annotations, and comparisons of the ICTV species demarcation criteria. Download Data Set S5, XLSX file, 0.02 MB. Copyright © 2021 Kawasaki et al. 2021 Kawasaki et al. https://creativecommons.org/licenses/by/4.0/ This content is distributed under the terms of the Creative Commons Attribution 4.0 International license.

### Phylogenetic analyses.

Multiple sequence alignments (MSAs) of picornaviral P1 nucleotide sequences for Fig. 5A, hepeviral ORF1 amino acid sequences for Fig. 5B, picornaviral 3D nucleotide sequences for Fig. 5D, and flaviviral NS5 nucleotide sequences for Fig. 5E were obtained from the ICTV resources (the family *Picornaviridae* [https://talk.ictvonline.org/ictv-reports/ictv_online_report/positive-sense-rna-viruses/picornavirales/w/picornaviridae/714/resources-picornaviridae], the family *Hepeviridae* [https://talk.ictvonline.org/ictv-reports/ictv_online_report/positive-sense-rna-viruses/w/hepeviridae/731/resources-hepeviridae], and the family *Flaviviridae*: [https://talk.ictvonline.org/ictv-reports/ictv_online_report/positive-sense-rna-viruses/w/flaviviridae/371/resources-flaviviridae]). Because an astroviral MSA was not available in the ICTV resources, we extracted astroviral ORF2 amino acid sequences from the RefSeq protein viral database (version 20201007). The MSAs of reference and novel viral sequences were constructed by MAFFT with the options “–add” and “–keeplength”. The astroviral MSA was trimmed by excluding sites where >20% of the sequences were gaps and subsequently removing sequences with less than 80% of the total alignment sites. Phylogenetic trees were constructed by the maximum likelihood method using IQ-TREE (version 1.6.12) (56). The substitution models were selected based on the Bayesian information criterion provided by ModelFinder (57): GTR+R8 for Fig. 5A, LG+F+R4 for Fig. 5B, LG+F+R5 for Fig. 5C, TVM+R9 for Fig. 5D, and GTR+R7 for Fig. 5E. The branch supportive values were measured as the ultrafast bootstrap by UFBoot2 (58) with 1,000 replicates. Tree visualization was performed by the ggtree package (version 2.2.1) (59). Sequence accession numbers used for the phylogenetic analyses are listed in Data Set S5.

### Comparison with the ICTV species demarcation criteria.

To assess whether the viruses identified in this study could be assigned to a novel species, we compared their genetic distances with those of known viruses according to the ICTV species demarcation criteria (34, 35) (Fig. S2). Amino acid sequences of the P1 and 2C + 3CD regions in hepatoviruses and parechoviruses were extracted by referring to those of hepatovirus A (GenBank accession no. M14707.1) and parechovirus A (GenBank accession no. S45208.1), respectively. Amino acid sequences of the NS3 and NS5B regions in pegiviruses were extracted by referring to those of pegivirus A (GenBank accession no. U22303.1). We constructed MSAs using these reference and novel viral sequences by MAFFT with the option “–auto”. We did not analyze other viruses identified in this study, because the ICTV did not provide criteria based on genetic distance. The sequence accession numbers used for these analyses are listed in Data Set S5.

### Mapping analyses using viral genomes identified in this study.

To verify the quality of sequence assembly, we mapped the reads in the RNA-seq data in which a novel viral sequence was identified to the viral genomes by STAR (version 2.7.6a) (60) (Fig. 4). The genome indexes were generated with the option “–genomeSAindexNbases” according to each viral genomic size, and mapping analysis was conducted with the option “–chimSegmentMin 20”. The number of mapped reads in each position was counted by Bedtools genomecov (version 2.27.1) (61) with the options “-d” and “-split”.

To identify novel viral infections in other individuals, we analyzed the publicly available RNA-seq data of the host animals by quantifying viral reads (Fig. 6A, B, and E). We investigated 1,593 goat, 91 blind mole-rat, 4 galago, 8,282 cow, and 59 tree shrew data for infections of goat hepatovirus, blind mole hepevirus, galago hepevirus, bovine parechovirus, and tree shrew pegivirus, respectively. Mapping analyses were performed using STAR (version 2.7.6a) as described above. The numbers of total and mapped reads were extracted by Samtools (version 1.5). We considered that there was a viral infection in the sample if the RPM was >1.0.

We compared the viral read amounts between the patient and control monkeys to investigate the association between chronic diarrhea and MLB-like astrovirus infection (Fig. 6D). Viral read amounts were quantified as described above. The average RPM for each individual is plotted in Fig. 6D, because six samples were collected from each individual. Data Set S6 shows the SRA run accession numbers used to investigate novel viral infections. Data Set S7 list sample metadata in which the novel viral infections were detected.

10.1128/mBio.01638-21.8 DATA SET S6 Sequence Read Archive run accession numbers used for mapping analyses. Download Data Set S6, XLSX file, 0.2 MB. Copyright © 2021 Kawasaki et al. 2021 Kawasaki et al. https://creativecommons.org/licenses/by/4.0/ This content is distributed under the terms of the Creative Commons Attribution 4.0 International license.

### Comparison of hepeviral sequences identified in different blind mole-rats.

We compared nucleotide sequence identities among the hepeviral sequences found in five different individuals to predict when these viruses infected the blind mole-rats. The sequence comparison was performed by BLASTN (version 2.11.0) with default parameters. Because most hepeviral sequences were detected as short contigs, sequence identities were represented by the percentage of identical matches in the longest aligned region between the hepeviral sequences (Fig. 6C). We also analyzed the total aligned length between contigs identified in each individual and the hepeviral genome identified in SRA accession no. ERR1742977 and confirmed that these contigs covered from 86.0% to 99.9% of the blind mole-rat hepevirus genome.

### Data availability.

The relevant codes and data are available at https://github.com/Junna-Kawasaki/virome_2021 and in the Mendeley data repository (doi:10.17632/stscmh9mr3.1).
