# Supplementary figures and images for: Hidden Viral Sequences in Public Sequencing Data and Warning for Future Emerging Diseases
Source: mBio. 2021 Aug 17;12(4):e01638-21. doi: 10.1128/mBio.01638-21 (PMC8406186; doi:10.1128/mBio.01638-21)

# Supplemental Figure 1

**A**

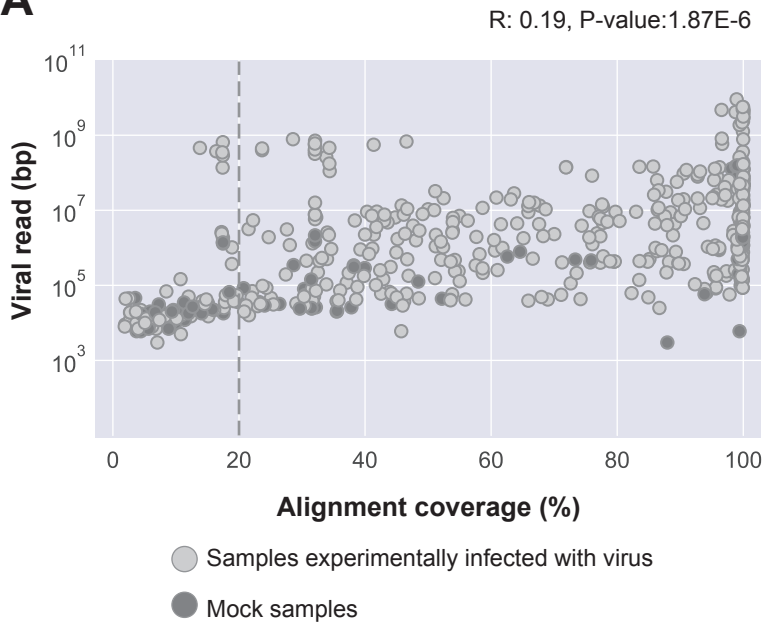

**B**

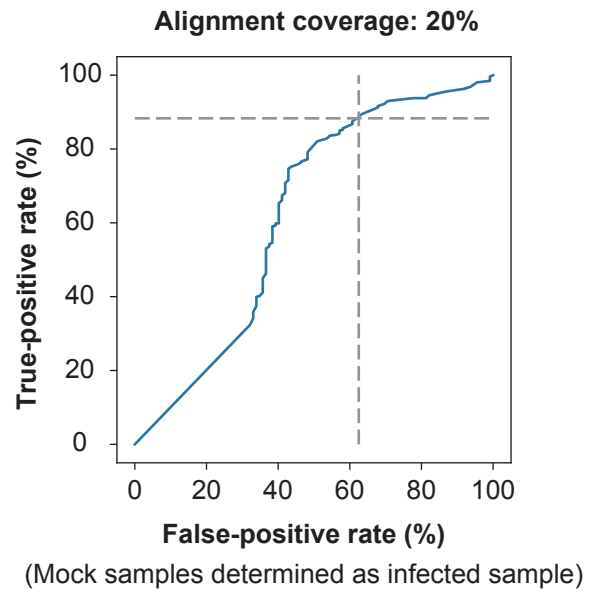

**C**

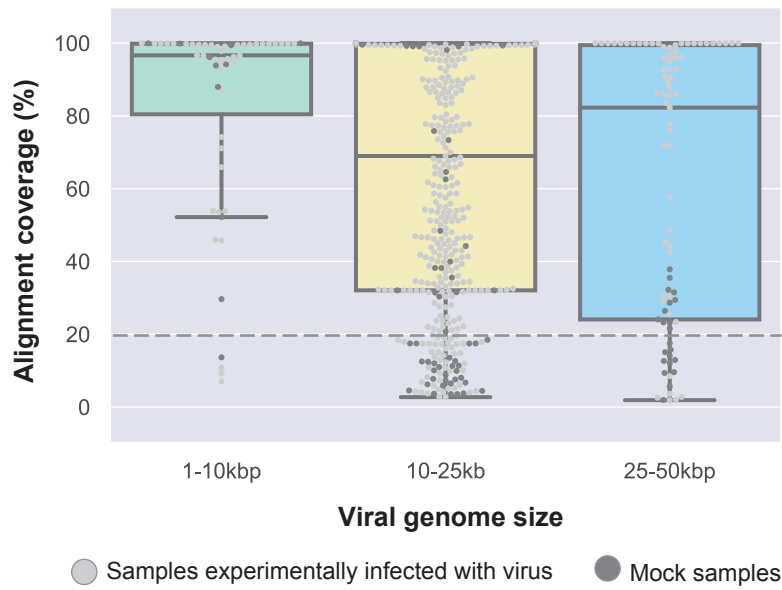

**D**

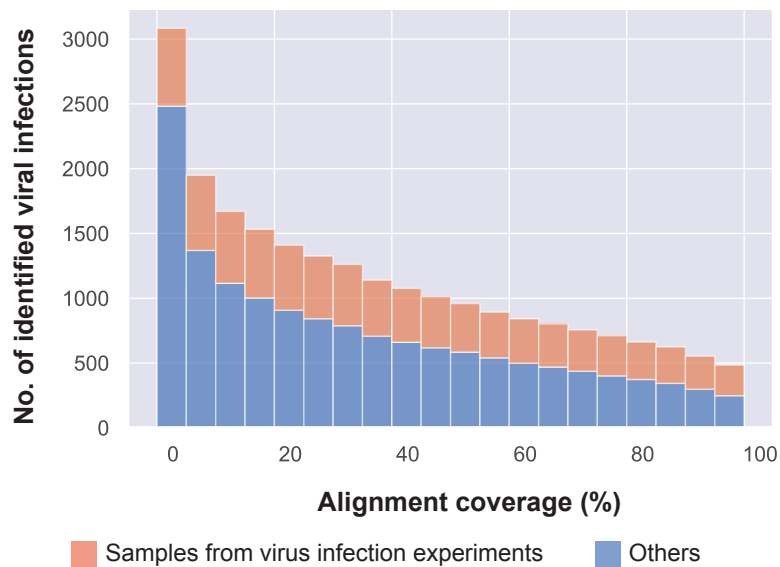

Supplement: FIG S1 [file mbio.01638-21-sf001.pdf]

# Supplemental Figure 2

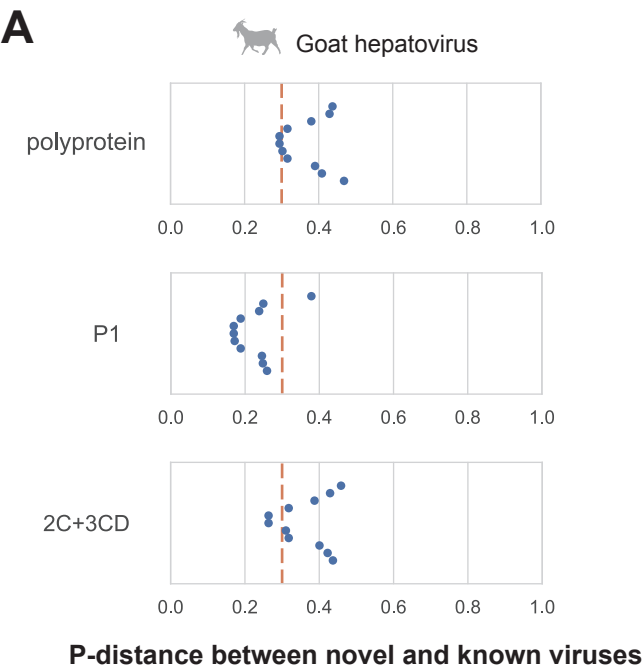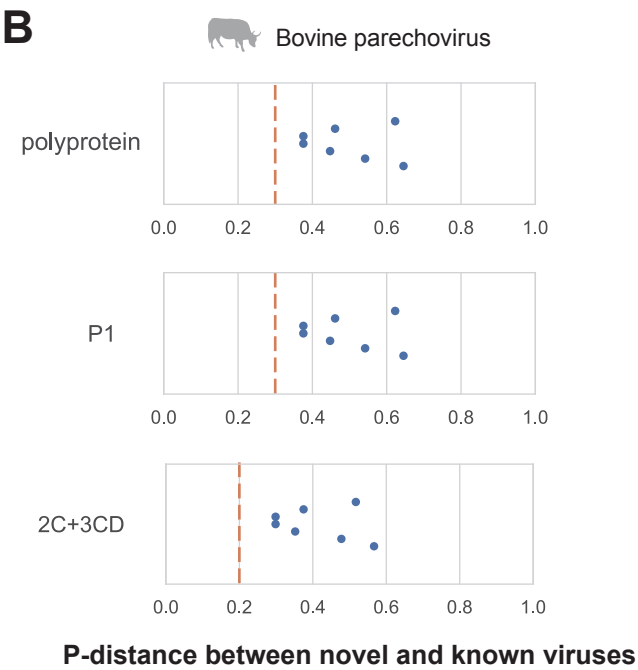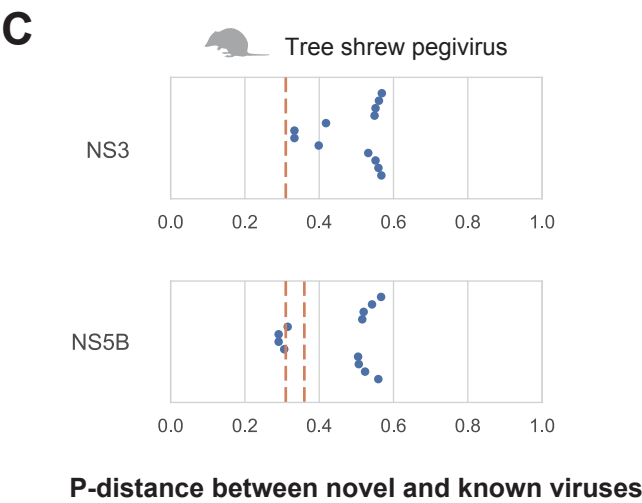

Supplement: FIG S2 [file mbio.01638-21-sf002.pdf]
